# Improving the Corrosion Performance of Organically Coated Steel Using a Sol–Gel Overcoat

**DOI:** 10.3390/ma17051075

**Published:** 2024-02-26

**Authors:** Evan Watkins, Chris M. Griffiths, Calvin A. J. Richards, Sarah-Jane Potts, Chris Batchelor, Peter Barker, Justin Searle, Eifion Jewell

**Affiliations:** 1 Faculty of Science and Engineering, Swansea University, Bay Campus, Crymlyn Burrows, Swansea SA1 8EN, UK; 2 Tata Steel UK, Shotton Works, Deeside CH5 2NH, UK

**Keywords:** sol–gel, coatings, corrosion, barrier, coil coating

## Abstract

Organically coated steels are widely used in applications in which they are subjected to the natural environment and therefore require excellent corrosion resistance. Organic clearcoats are typically employed as a barrier that improves the overall corrosion resistance; however, they are typically derived from fossil fuel-based feedstock. A more sustainable alternative could be possible using sol–gel coatings. The application of a simple tetraethoxysilane (TEOS)-based sol–gel was applied to polyurethane-coated steels using a spray coater. The concentration of TEOS was altered to produce coatings containing either 2.5% or 10%. The 10% TEOS resulted in dense, homogeneous coatings that offered a significant improvement in corrosion resistance compared to an uncoated substrate. Whereas the 2.5% TEOS coatings were inhomogeneous and porous, which indicated a limitation of concentration required to produce a uniform coating. The successful demonstration of using a simple TEOS-based coating to improve the corrosion resistance of organically coated steel highlights the potential for further investigation into the use of sol–gels for these applications.

## 1. Introduction

Organically coated hot-dipped galvanised (HDG) steels are widely used throughout the automotive and construction industries. Organic coatings provide improved corrosion protection to the underlying steel and offer a range of colours and textures for aesthetic purposes [1]. These coating systems are made up of several layers: a pretreatment to provide adhesion to the overlying primer, a primer that contains active corrosion inhibitor pigments and a topcoat that provides barrier protection to the external environment and is responsible for the aesthetics of the product [1,2]. A 15–20 µm thick clearcoat is also commonly applied to polyurethane (PU) coatings to improve barrier protection by providing improved resistance to the transportation of water, oxygen and aggressive ions such as chloride [2,3]. The migration of aggressive ionic species through the coating to the metal substrate can lead to coating delamination and underfilm corrosion, which will limit the lifetime of the material. The uptake of water to a coating is an important consideration as it facilitates the transport of oxygen and ionic species; thus, improving coating resistance to water uptake can improve the corrosion performance [4,5,6,7]. However, although organic coatings provide some resistance to water, oxygen and ionic species, they are susceptible to degradation, and corrosion readily occurs when exposed to external environments [6,8,9,10]. Moreover, the use of a typical clearcoat requires the application of additional material and the subsequent extended curing times in gas-powered ovens [2]. This increases both the cost and the carbon footprint required to produce the finished organically coated product.

Sol–gel coatings are thin (<5 µm), dense, ceramic-like coatings that offer the benefits of being non-toxic, low cost, simple formulation and the ability to add functional properties such as hydrophobicity, improved hardness and scratch resistance [11,12,13,14]. These coatings are formed through the hydrolysis and condensation reactions of alkoxide precursors within an organic solvent that form layers of silicon oxides when cured onto a surface. The most extensively studied alkoxide precursors are silane-based as they are low cost, widely available and are of lower reactivity compared to other alkoxide precursors based on alumina, titania and zirconia [15,16,17]. Sol–gel coatings are formed in four stages: hydrolysis, condensation, chain growth and agglomeration [18]. Once the sol has been prepared, hydrolysis and condensation reactions take place, which produce alcohol and water, as shown in Reactions (1–3). The properties of the coatings produced through these reactions can be modified by changing the solution pH, temperature, stoichiometry and the inclusion of additives such as silicon dioxide [19,20,21]. Recent research has also demonstrated that sol–gel coatings can be produced using bio-composites and green chemistries, reducing the carbon footprint required to manufacture the coatings [22,23].

(1) $$\equiv Si-OR+H_{2}O⇋\equiv Si-OH+ROH$$

(2) ≡Si−OR+HO−Si≡⇋≡Si−O−Si≡ROH

(3) $$\equiv Si-OH+HO-Si\equiv ⇋\equiv Si-O-Si\equiv +H_{2}O$$

Sol–gel coatings have been applied to a range of metallic substrates as a pretreatment to improve corrosion resistance by forming a barrier layer [19,24,25]. However, during the formation of sol–gel coatings, cracks and pores can form due to the high stresses that occur within the coating during curing, which limits the corrosion resistance [13,19,26]. Research has investigated the inclusion of corrosion inhibitor pigments to the sol–gel coatings to provide active corrosion protection [26,27,28,29,30,31]. The use of sol–gel coatings as a pretreatment has also been reported to improve the adhesion of organic coatings through the formation of strong covalent Me-O-Si bonds during the drying stage [25].

Another area of interest involving sol–gel coatings is that of the enhancement of organic coatings. Yu et al. reported that a sol–gel system mixed with silicon dioxide and waterborne polyurethane produced an effective corrosion protective coating for a 55% Al-Zn alloy-coated steel [32]. The results indicated that the combined sol–gel PU coating outperformed commercial chromate and chrome-free conversion coatings when subjected to salt spray testing. Moghaddam et al. reported that an acrylic-based clearcoat containing tetraethyl orthosilicate (TEOS) and methacryloxy popyltrimethoxysilane (MEMO) improved the mechanical properties of the clearcoat without affecting its appearance or transparency [33].

Although sol–gel coatings have been well studied, there is limited published work on their use as an overcoat on an organic substrate. Typical organic clearcoats are thick (~15 µm) but offer limited corrosion protection [2]. In comparison, sol–gel coatings are thinner, can improve the corrosion performance on metallic surfaces, offer improved hardness and scratch resistance and provide hydrophobic properties. Recently published work demonstrated that sol–gels offer a potential replacement for organic clearcoats as they improved the mechanical and UV degradation performance whilst being substantially thinner [34]. The work demonstrated that TEOS-based sol–gel coatings can be applied to PU substrates, but further work was required to determine whether there is scope for industrial applications.

In the research presented here, a simple TEOS-based sol–gel formulation is sprayed and cured onto a PU-coated hot-dip galvanised steel substrate. The TEOS ratio within the coating formulation is altered to produce a 2.5% or 10% silicon oxide yield and is applied to substrates using a spray coater at three different head speeds to produce varying coating thicknesses. The gas barrier performance of the coatings is investigated via in situ FTIR within a novel closed-loop reactor and the corrosion performance is scrutinised by subjecting the coated samples to salt spray testing.

## 2. Materials and Methods

### 2.1. Sol–Gel Preparation and Application

The sol–gel was produced using tetraethoxysilane (TEOS) as the precursor, 1 M hydrochloric acid (HCl) as the catalyst and isopropyl alcohol (IPA) as the solvent. All chemicals were purchased from Sigma Aldrich (Burlington, MA, USA) and were of analytical grade. A stock solution was prepared by mixing TEOS, IPA, H_2_O and HCl at a mol ratio of 1:5.33:4:0.006. The TEOS was slowly added to the IPA whilst magnetically stirred. The water, acidified with the HCl, was added dropwise whilst magnetically stirred vigorously. The solution was sealed in a beaker and stirred for 2 h before being used. The stock solution was then diluted with IPA to produce final solutions of either 10% or 2.5% silicon oxide yield. Samples produced using 2.5% and 10% silicon oxide yield are referred to as 2.5% TEOS and 10% TEOS, respectively.

The coatings were deposited using a Sono-Tek ExactaCoat system, which deposited the coating using an ultrasonic spray head at a constant flow rate of 2.5 mL/min. The spray area was kept constant at 270 cm^2^ and the spray head speed was altered to produce varying dosages. Coated samples were placed into a convection oven at 200 °C for 10 min with no thermal ramp applied. These processing parameters were chosen to mimic the conditions that occur on a roll-to-roll coating line whereby the substrate would be heated within an inline oven system. Once cured, the sol–gel-coated substrates were removed from the oven and left to cool to room temperature. The samples created for this work are given in Table 1.

### 2.2. Scanning Electron Microscopy (SEM)

The microstructure of the prints was assessed using a JEOL JSL 7800F Field Emission Gun Scanning Electron Microscope (Tokyo, Japan) (FEG-SEM) (4 kV acceleration voltage, a working distance of 4 mm and magnifications of ×5000 and ×10,000). Cross-sections were prepared using ion beam milling (IM4000 Plus, ion milling system, for 1.5 h, acceleration voltage 4 kV, discharge voltage 1.5 kV) (Hitachi High-Tech America, Inc., Hillsboro, OR, USA) and cross-section samples were sputter-coated with 3 nm platinum.

### 2.3. White Light Interferometry

White light interferometry (NT9300, Veeco Instruments, Inc., Plainview, NY, USA) was used to measure a full three-dimensional surface profile of the coated films. Five-times magnification was used, giving a measurement area of 1.2 mm by 0.93 mm (at a resolution of 736 × 480 pixels with sampling at 1.67 µm intervals). Average surface roughness measurements (Ra) were measured across the entire measurement area and away from edges that were masked with tape.

### 2.4. Salt Spray Testing

The salt spray test (SST) is a standardised and widely accepted method of determining the corrosion resistance of coated metal substrates. The 2-layer polyurethane-coated hot-dipped galvanised steel samples were provided by TATA Steel Europe, which incorporated a 25 µm primer coating and a 25 µm topcoat. Samples were prepared in duplicate and were cut to 15 cm × 21 cm before being spray-coated using the settings shown in Table 1. Scribes were applied to the coated surface in an X pattern using a 0.5 mm diameter Erichsen Van Laar scribe. The top and side cut edges were covered by black PVC tape and were prepared as per ISO 17872:2019 [35]. The test was carried out in an SST chamber constructed according to ASTM B-117 [36] where the samples were exposed on racks angled at 45°. An aqueous solution of 5 wt.% NaCl (pH 7) was atomised to form a corrosive atmosphere and the chamber was held at 35 ± 2 °C for the duration of the exposure. Samples were tested in duplicate and compared to an uncoated PU substrate. Following the 1000 h exposure, the samples were removed from the cabinet, rinsed in deionised water and left to dry. Once dry, the cut edge and scribe creep areas were scrutinised and any delaminated paint manually removed using a flat metal instrument, taking care not to damage any non-delaminated paint. Photographs of the samples were taken using a Canon EOS 5D camera.

### 2.5. O_2_ Permeation Measurements Using FTIR in a Closed-Loop Flow Reactor

Previous work by Searle et al. demonstrated that a polyvinyl chloride organic coating containing TiO_2_ will photodegrade when subjected to UV-A radiation and that the breakdown of the coating could be monitored by measuring the CO_2_ released during this process [37]. The authors used a novel flat panel reactor attached to an FTIR, which facilitated the measurement of the time-dependent CO_2_ concentration. The work described here uses the same method to investigate the ability of sol–gel coatings, described in Table 1, to limit the diffusion of O_2_ when applied to a model organic coating containing TiO_2_ when subjected to UV radiation. The direct photolysis and subsequent oxidation of the polymer to CO_2_ is reliant on the availability of O_2_ at the site of degradation. The concentration of evolved CO_2_ will be rate limited by the availability of local O_2,_ which is affected by the sol–gel coating applied to the PVB substrate. Any defects in the sol–gel coating would contribute to higher concentrations of measured CO_2_ whereas a more homogeneous and defect-free coating would provide improved resistance to O_2_ permeation, resulting in a lower concentration of CO_2_.

**Figure 1:**
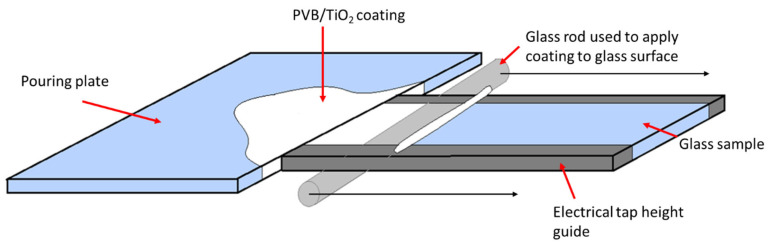
Schematic of sample preparation for use in the FTIR closed-loop flow reactor.

A model photocatalytic coating was prepared using titanium dioxide (P25, Degussa, Frankfurt, Germany), polyvinyl butyral-co-vinyl alcohol-co-vinyl acetate (PVB), molecular weight 70,000–100,000 and ethanol (≥99%, Sigma Aldrich). PVB was mixed with ethanol at 15.5 wt.% using a high-speed shear mixer for half an hour or until the PVB was fully combined. TiO_2_ (40 wt.%) was slowly added to the mixture whilst continuously shear-mixed until fully dispersed. The TiO_2_ films were coated using a draw down method onto borosilicate glass panels (300 mm × 150 mm × 2 mm). Electrical PVC tape was applied to the long edges of the glass, which acted as a height guide for the coating that was applied using a glass rod, shown schematically in Figure 1. The coating was air-dried for 24 h before being spray-coated with the sol–gel coatings shown in Table 1.

**Figure 2:**
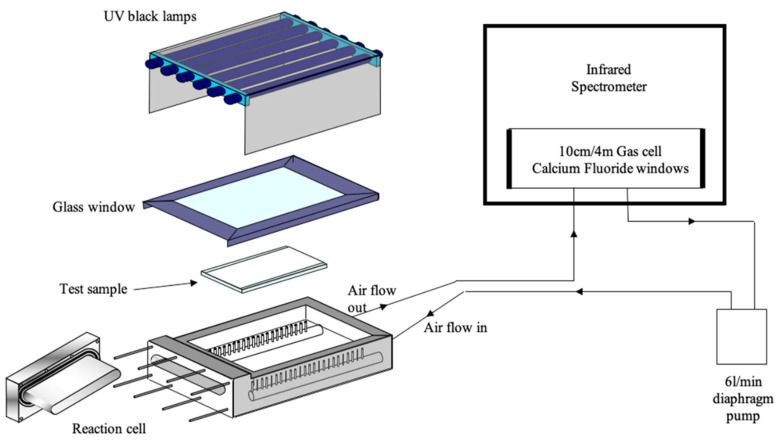
Schematic of the FTIR closed-loop flow reactor [38].

A full description of the design and operational working of the flat panel reactor and continuous FTIR monitoring technique has been given elsewhere and a schematic of the design is shown in Figure 2 [38,39]. In brief, the reactor consists of a solid polycarbonate frame with 5 mm diameter holes drilled at each end. A borosilicate glass sheet is clamped on one side of the frame and the coated sample is clamped, coating side down, onto the other side with rubber seals on both sheets to ensure an airtight seal and allows the coating to be irradiated by an external source. The UV irradiation source consists of 6 × 8 W UV lamps (λ_max_ 365 nm), which were placed 5 cm above the uncoated borosilicate glass. The frame of the reactor was connected to a diaphragm pump using Masterflex tubing (TYGON R-3606), which circulates the gas in the sealed system that passes through an infrared flow cell (path length 10 cm) mounted inside a Fourier-transform infrared spectrometer (FTIR Perkin Elmer FT100, Walthman, MA, USA). The whole system has a volume of ~770 cm^3^.

**Figure 3:**
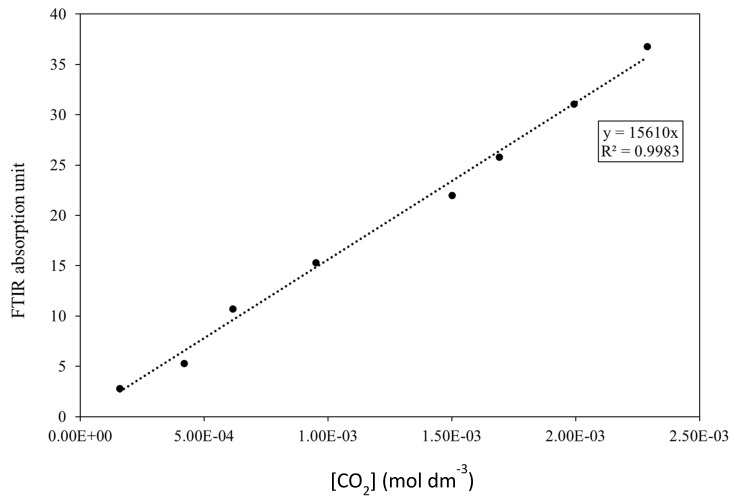
Calibration plot showing the FTIR signal response with varying concentration of injected CO_2_.

The closed system is comprised of air and CO_2_ was the primary gas measured during this experiment, which is naturally present in the atmosphere and can be affected by laboratory occupants present in the vicinity of the FTIR. Background subtraction was therefore performed to ensure that any variation in CO_2_ concentration within the system was measured accurately. To measure CO_2_ concentration, the area integration of the associated FTIR peak in the region 2200–2500 cm^−1^ was measured over time [38,39]. The response of the FTIR to increasing CO_2_ was calibrated by injecting known amounts of CO_2_ into the system using a syringe. The FTIR signal is recorded after each addition and a calibration curve produced by plotting these data is shown in Figure 3.

### 2.6. ATR-FTIR

The chemical structure of the sol–gel-coated samples was characterised via attenuated total reflectance Fourier-transform infrared spectroscopy (ATR-FTIR) using a Perkin Elmer FT100 with a Specac ATR attachment. Spectra were collected in transmittance mode with 8 scans at a 4 cm^−1^ resolution in the wavelength range of 4000–500 cm^−1^ and data were recorded and analysed using Spectrum 10.5.4 software.

## 3. Results

### 3.1. Coating Morphology and Thickness

**Figure 4:**
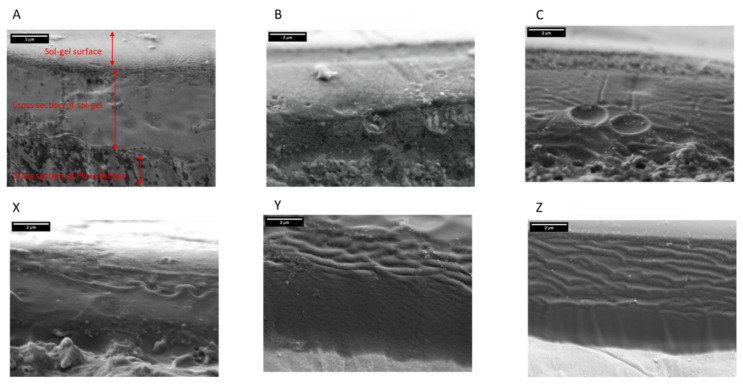
FEG SEM cross-sectional images produced by ion beam milling. Images taken at 10,000× magnification (scale bar = 2 μm). The images show the sol–gel surface, cross-section of the sol–gel coating and cross-section of the PU substrate; 2.5% Si yield (**A**–**C**) and 10% Si yield (**X**–**Z**).

SEM micrographs of ion beam-milled cross-sections of the sol–gel-coated PU samples are shown in Figure 4. The structure of the coating is shown in Figure 4A with the coating surface, and cross-section of the sol–gel and substrate marked. The morphology of the cross-sections seen in Figure 4 varies with both the silicon concentration and dosage. Studying the cross-sectional images for samples A–C shows that A has a dense uniform sol–gel layer but that B and C show evidence of pores and defects throughout the cross-section. Sample C has large voids within the coating cross-section and pores are evident at the sol–gel–PU interface. In comparison, the cross-section of the 10% TEOS samples (X–Z) all show that the sol–gel coating has no visible porosity and has a homogeneously dense structure. The average coating thickness is obtained from five measurements taken across the cross-section of the sol–gel coating and are given in Table 2. Coatings produced using the 2.5% TEOS had average coating thicknesses between 4.6 and 5.1 µm and those produced using the 10% TEOS had thicknesses of 4.5 to 5.8 µm. Samples B and C had a larger variation in coating thickness when compared to the other coatings, which indicates heterogeneity. Overall, the samples produced using the 10% TEOS were thicker than the samples produced using the 2.5% TEOS.

**Figure 5:**
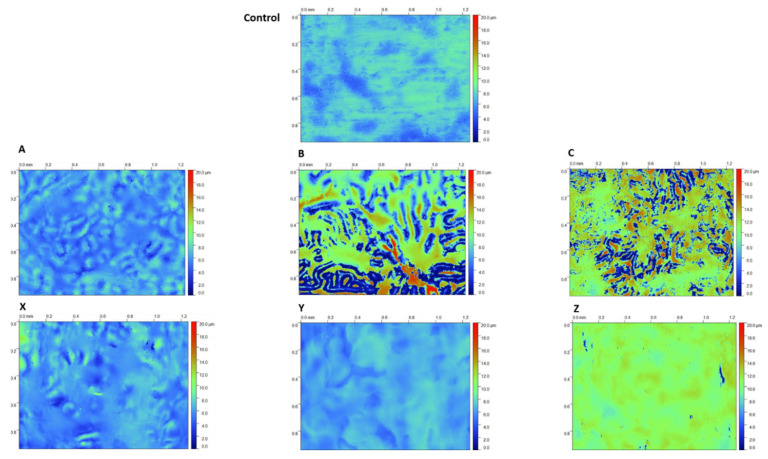
White light interferometer maps of a bare PU-coated steel surface (control) and sol–gel-coated PU substrates; 2.5% Si yield (**A**–**C**) and 10% Si yield (**X**–**Z**).

Coating topography was characterised using white light interferometry (WLI), which is presented in Figure 5 with the corresponding surface roughness values given in Table 2. The control sample shown in Figure 5 is a bare PU-coated steel surface, which is observed to have small changes in topography highlighted by blue and green colours and an average roughness of 0.75 µm. The application of the 2.5% and 10% TEOS coatings has an observable effect on the surface roughness. The topography of sample A is slightly smoother than that of the control; however, samples B and C have a significantly different coating topography. Sample B shows that there is a high variation in the surface topography with dark blue and reds visible in areas of the map and the surface roughness measurement of 3.69 µm, which is the roughest of all samples. The WLI map in Figure 5 shows that the surface has formed an island-like structure with areas of peaks surrounded by valleys. Sample C also has an inhomogeneous surface topography, shown in Figure 5, and a surface roughness of 3.1 µm, which is considerably rougher than that of the control. In comparison, the coating produced by the 10% TEOS produced smoother surfaces compared to those of the control. The WLI maps show that these samples produce consistent, relatively homogeneous coatings compared to those of the 2.5% TEOS. The surface roughness data, Table 2, show that these coatings are smoother than those of the uncoated PU. There are small areas of dark blue observed on sample Z, which may be evidence of coating defects such as cracks or pinholes.

### 3.2. Salt Spray

**Figure 6:**
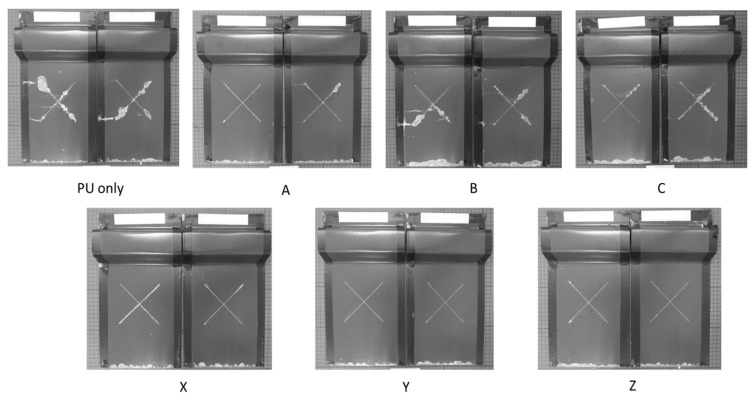
Optical images of a PU sample and sol–gel-coated PU samples after 1000 h salt spray exposure (5% NaCl); 2.5% Si yield (**A**–**C**) and 10% Si yield (**X**–**Z**).

**Figure 7:**
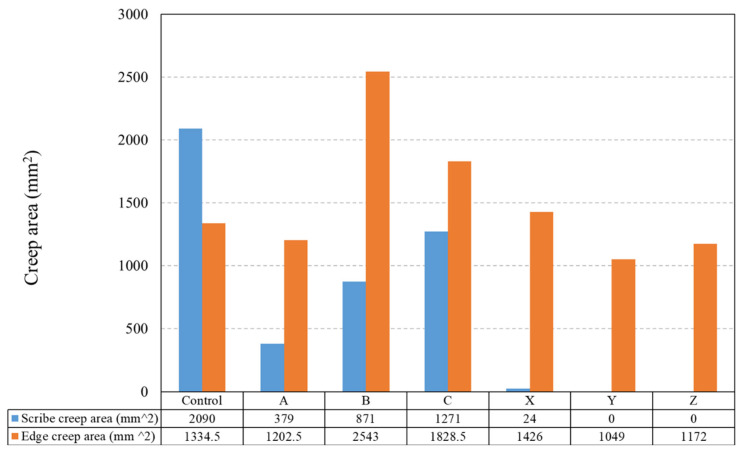
The edge and scribe creep area (mm^2^) observed on sol–gel-coated PU samples after 1000 h salt spray exposure (5% NaCl).

The corrosion performance of the sol–gel-overcoated PU samples was evaluated via salt spray testing with a 5% sodium chloride (NaCl) solution for 1000 h. Photographs of the samples post-testing are shown in Figure 6. Coating disbondment at the cut edge and scribe were measured using photo-editing software (Image-Pro, Media Cybernetics, Rockville, MD, USA), which calculated the area of bare metal visible compared to the intact paint. Measurements were taken on both samples for each coating with an average taken and plotted in Figure 7.

The measured delamination, given in Figure 7, shows that the worst-performing samples were those of the PU coating with no sol–gel overcoat with an average of 2090 mm^2^ scribe creep and 1334.5 mm^2^ edge creep over the two samples. All sol–gel-coated samples observed a significant reduction in the scribe creep area with samples Y and Z reporting no delamination at all. The poorest-performing sol–gel coating, C, had a measured scribe creep area of 1271 mm^2^, which is a reduction of 39% compared to the uncoated PU control. The edge creep performance is shown to be more variable across the sol–gel-coated samples. The best-performing sample, Y, observed an edge creep area of 1049 mm^2^, which is a 21% reduction when compared to the uncoated PU control. However, three samples, B, C and X, all observed an increase of 91%, 37% and 7% in the edge creep area when compared to the uncoated PU control. Studying Figure 6 and Figure 7, the coatings produced using the 10% TEOS (X–Z) had less edge and cut edge creep than those of the low 2.5% TEOS coatings (A–C).

### 3.3. O_2_ Barrier Measurements

**Figure 8:**
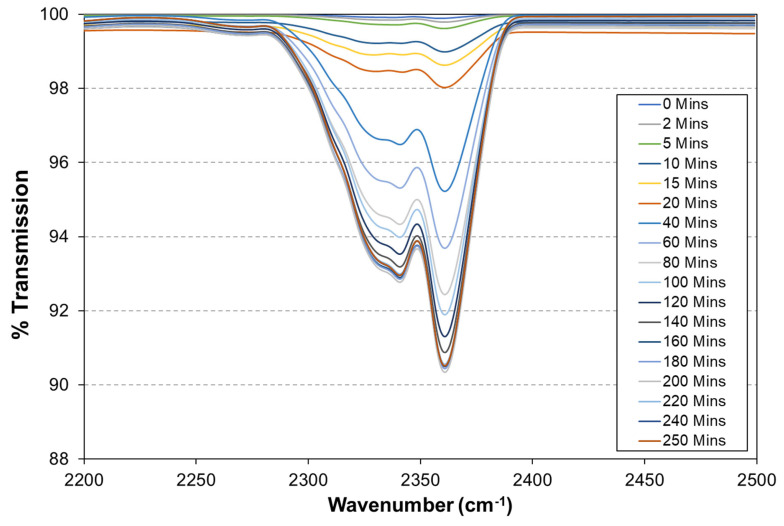
The time-dependent change in FTIR peak between 2200 and 2500 cm^−1^ for a PVB-coated glass sample containing 40 wt% TiO_2_.

The O_2_ permeability of the sol–gel coatings was determined by using a novel closed-loop reactor that incorporates an FTIR to measure the time-dependent increase in CO_2_ concentration that occurs via the UV degradation of PVB coatings containing 40 wt.% TiO_2_. The time-dependent release of CO_2_ when PVB-coated glass is exposed to UV is used as a control and the resultant FTIR transmission (%) peak between 2200 and 2500 cm^−1^ is shown in Figure 8. There was an observed decrease in transmission over time, which represents the increase in CO_2_ concentration within the closed-loop reactor as the PVB coating is irradiated.

**Figure 9:**
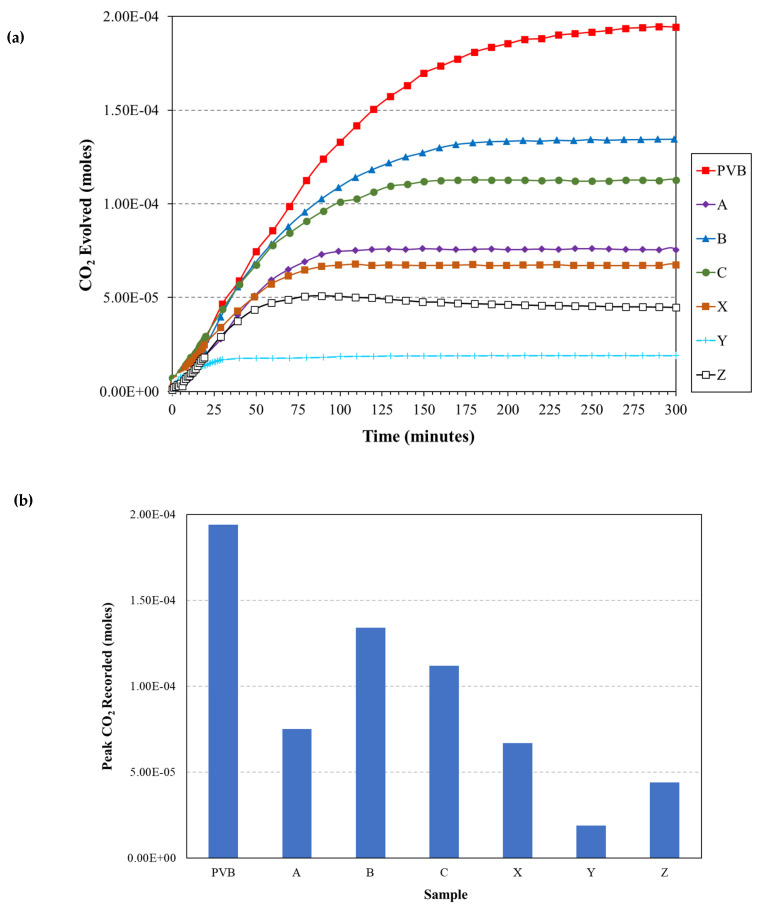
(**a**) The time-dependent change in CO_2_ recorded for UV-irradiated sol–gel-sprayed PVB-coated glass samples using a closed-loop FTIR flat panel reactor. (**b**) Peak CO_2_ measured for each sample over the 300 min flat panel reactor experiment.

Figure 9a shows the change in CO_2_ concentration recorded using FTIR for the sol–gel-coated PVB samples irradiated with UV within the closed-loop reactor over 300 min. The 300 min duration of the experiment was chosen due to no further increase in CO_2_ concentration being measured over longer durations. The corresponding maximum CO_2_ concentration produced by each sample is given in Figure 9b. The PVB control sample, having no sol–gel coating, produced the highest concentration of CO_2_ within the closed-loop reactor. This shows that all sol–gel coatings impede O_2_ transport through the coating to the PVB and therefore a reduction in the observed evolved CO_2_. The sol–gel coating containing the 2.5% TEOS recorded higher concentrations of CO_2_ compared to the 10% TEOS sol–gel coatings. The lowest concentration of CO_2_ recorded was provided by sample Y at 1.9 × 10^−5^ M of CO_2,_ which is a reduction of 90% compared to the CO_2_ recorded from the control.

### 3.4. Contact Angle

**Figure 10:**
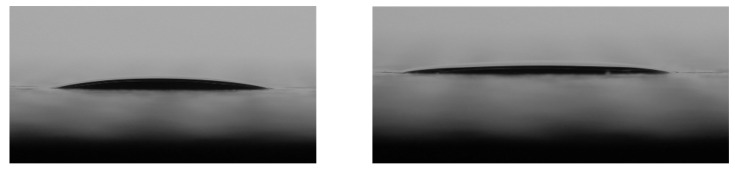
Contact angle images showing a droplet of (**left**) 2.5% Si solution and (**right**) 10% Si solution on a polyurethane coating. The CA measurements were calculated to be 13° and 9°, respectively.

The contact angle (CA) provides a quantitative measure of the wettability of the sol–gel solution on the PU surface. CA measurements were obtained using a goniometer (Ossila) with an accuracy of ±1° under optimised illumination and optical conditions. Figure 10 shows the contact angle of the 2.5% TEOS and 10% TEOS coatings when applied to a PU-coated steel surface. The CA measurement of the 2.5% TEOS solution was recorded to be 13° and the 10% TEOS was recorded as 9°. These low contact angles indicate that the solutions both achieve good wetting of the PU surface.

### 3.5. ATR-FTIR Analysis

**Figure 11:**
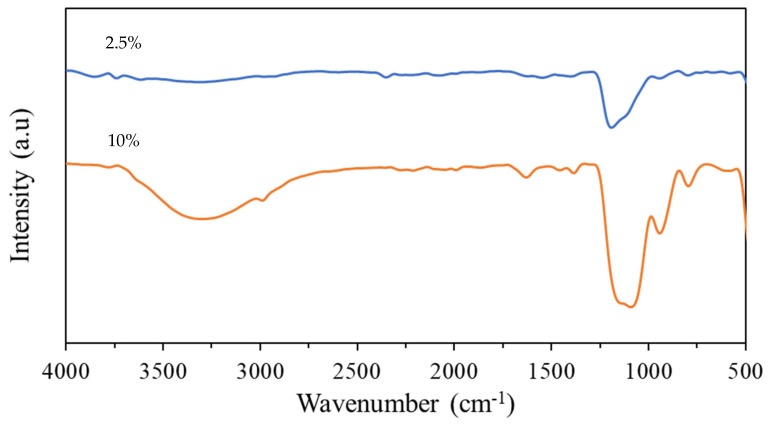
FTIR spectra of 2.5% and 10% TEOS coatings.

The effect of the TEOS concentration on the chemical composition and bond characteristics of the resultant sol–gel coatings was confirmed using ATR-FTIR. The infrared spectra of the cured 2.5% and 10% TEOS coatings is shown in Figure 11. The 10% coating contained a strong broad peak between 3100 and 3500 cm^−1^ due to the presence of O–H species such as the hydrolysis product Si–OH and remaining H_2_O within the coating [40]. The presence of water within the coating was confirmed by the small peak at ~1630 cm^−1,^ which corresponds to O–H bending vibrations. A strong broad band between 1200 and 1000 cm^−1^ in both the 2.5% and 10% coatings can be attributed to Si–O–Si vibrations. It has been shown that the peaks at ~1070 cm^−1^ and ~1200 cm^−1^ correspond to the transverse optical and longitudinal optical antisymmetric stretches, respectively, for strained fourfold siloxane rings [41]. The 10% TEOS coating contained two additional peaks at ~950 cm^−1^ and ~790 cm^−1^. The peak at 950 cm^−1^ corresponds to Si–OH bending and the peak at 790 cm^−1^ corresponds to Si–O–Si bending vibration [42]. The presence of only the Si–O–Si vibration for the 2.5% TEOS coating was unexpected based on previous works [34,40,41]. The presence of all the expected peaks in the 10% TEOS coatings suggests that polysiloxane network formation has occurred at this concentration. However, for the 2.5% TEOS coating, the missing peaks and weaker Si–O–Si stretch suggest the coating network formation is poor. This suggests there was a concentration limit of sufficient Si species remaining in the coating to undergo hydrolysis and condensation reactions to form a homogenous film.

## 4. Discussion

A TEOS-based sol–gel coating was used to overcoat PU-coated steel substrates. The TEOS concentration was varied to provide a 2.5% or 10% silicon oxide yield. The coating was deposited using a spray-coating system, varying the head speed to produce changes in the coating morphology and thickness.

The coating thickness was characterised using FEG-SEM to image the cross-section of the sol–gel-coated PU samples, as shown in Figure 4 and measurements given in Table 2. The images show that there are significant differences in the coatings produced using the 2.5% Si or 10% TEOS coatings. The coatings produced using 10% Si are homogenous, dense and have no visible defects such as pores or cracks. Sample A, produced using the 2.5% TEOS, shows little porosity and is dense. However, samples B and C both show signs of porosity with pores observed at the coating–PU interface for sample B and large pores throughout the sol–gel coating for sample C. Samples B and C were produced using larger dosages of the coating compared to that of A, which would typically be associated with a thicker coating, but this was not observed. The WLI data, given in Figure 5, show the surface morphology of the sol–gel-coated PU surfaces. Studying these images, it is clear that samples B and C have a much different surface morphology when compared to all other samples. Here, there are large islands of coating that sit proud of the underlying substrate, producing a very rough and inhomogeneous surface topography. These features are only observed in the 2.5% TEOS samples, which indicates that the low TEOS concentration is the cause. The mechanism of coating formation for sol–gels is through the condensation of Si–OH with –OH species on the substrate surface, which makes them ideal for use on metallic surfaces. However, polymeric surfaces have limited sites for this reaction to initiate and therefore are more challenging to produce sol–gel coatings on [43]. The 2.5% TEOS may be too dilute to allow for a consistent number of Si–OH bonds to form a homogenous coating. The surface morphology observed in the WLI for samples B and C is similar to that of the coffee-ring effect that may occur during the curing process of the sol–gel [44]. Due to the low concentration of TEOS in the 2.5% Si coating, adhesion at the interface via condensation between the sol–gel coating and PU substrate is limited, resulting in regions that are unable to form a homogenous coating. The limited concentration gives rise to the formation of island-like structures of coating to form where areas of high Si concentration form a thick film surrounded by areas of no or little coating. This is also shown in the surface roughness data presented in Table 2 whereby both samples B and C (2.5% TEOS) are up to five times rougher than those of other sol–gel coatings.

**Figure 12:**
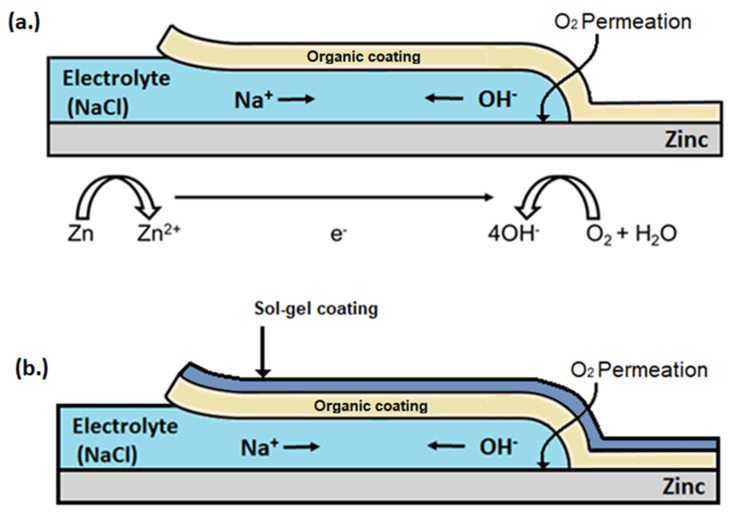
Schematic of the cathodic disbondment mechanism responsible for the coating failure observed in Figure 9 with (**a**) polyurethane-coated HDG steel sample and (**b**) a sol–gel-sprayed polyurethane-coated HDG steel sample.

The salt spray data show that all sol–gel coatings provide superior corrosion protection compared to a bare PU substrate. Coatings produced using the 10% TEOS performed the best with a small amount of scribe creep observed for sample X, produced with the lowest dosage of 1.11 µL/µm^2^. No scribe creep was observed for samples Y and Z, produced using a 2.22 and 4.44 dosage (µL/µm^2^), respectively. The dense, homogeneous sol–gel coatings produced provide an additional barrier to the external environment, which significantly improves the corrosion performance compared to an uncoated panel. All the 2.5% TEOS samples performed poorer compared to the 10% TEOS coatings, with scribe creep observed for all samples. These coatings were shown to be inhomogeneous and varied significantly in thickness across the surface. Coating failure at the scribe or edge occurs via the corrosion process of cathodic disbondment when an electrolyte is in contact with the bare metal/coating interface [45,46], which is shown in Figure 12a. Anodic metal dissolution occurs at the cut edge or defect, and the cathodic reaction occurs at the metal–organic coating interface with a layer of electrolyte linking the reactions [47,48]. The pH at the cathode is elevated (pH ≥ 10) due to OH^-^ formation, which leads to the disbondment of the coating via several different processes such as dissolution of the zinc (hydr)oxide layer to soluble zincate and via base-catalysed hydrolysis [46,47,49,50]. This corrosion mechanism can be slowed or stopped by inhibiting the anode or cathodic reactions, blocking ionic transfer in the electrolyte or electron transfer at the cathode. It is proposed here that the sol–gel coating provides an additional barrier for oxygen to permeate through, reducing the rate of reaction at the cathode and slowing the overall corrosion rate, shown in Figure 12b. This mechanism explains the poor performance of samples B and C due to the inhomogeneity of the coating creating areas of higher oxygen ingress rate and therefore poor corrosion performance. All samples observed edge creep; however, the worst-performing samples were B and C where an increase in edge creep is observed compared to that of the control. The cut edge is a worse case compared to the scribed area as there is a larger surface area for oxygen to permeate into and any corrosion-inhibiting mechanisms associated with the reduced oxygen permeation via sol–gel coatings are less impactful at these areas. Samples Y and Z had improved edge creep compared to the control; however, the performance is not as profound as that of the scribe creep.

**Figure 13:**
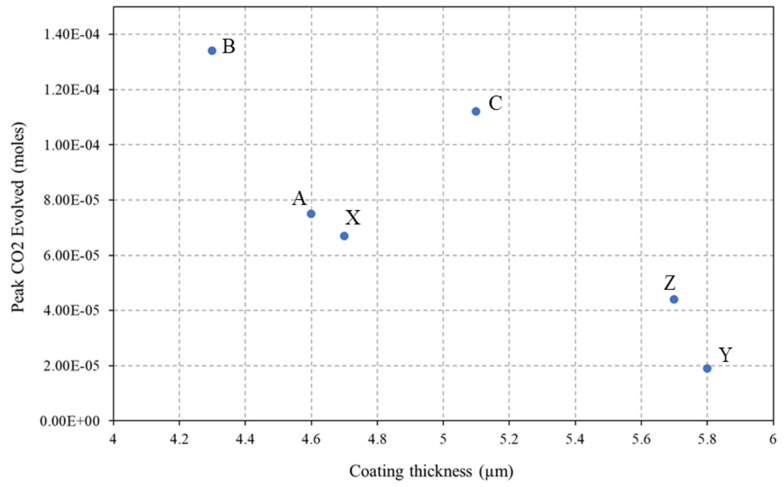
The peak CO_2_ concentration for varying sol–gel thicknesses. Data points are labelled with the corresponding sample I.D.

The sol–gel coating gas barrier performance was investigated using FTIR to measure the time-dependent release of CO_2_ from UV-irradiated sol–gel-coated PVB containing TiO_2_. The results, shown in Figure 10, demonstrated that the sol–gel coatings provide a barrier to CO_2_ compared to those of an uncoated sample. Coatings produced using the 2.5% TEOS performed worse than those using the 10% TEOS with the best-performing coating being sample Y. Sample Y had a dense, homogeneous coating with no visible defects in the WLI maps and was the thickest coating, shown in Table 2. The poorest-performing coatings were B followed by C, which were produced using 2.5% Si and were shown to have produced an inhomogeneous coating. Figure 13 shows the relationship between the peak evolved CO_2_ concentration and sol–gel coating. The general trend is that the thicker the sol–gel coating, the better the barrier performance. The outlier at 5.1 µm thickness is that of sample C, which was shown to have an inhomogeneous coating and larger variations in roughness. The inhomogeneous nature of this coating would allow for quicker CO_2_ permeation at areas of low coating formation and therefore produce the large concentrations observed in Figure 13. These results give further evidence that the sol–gel coatings are providing a gas barrier to the underlying PU substrates in the salt spray test whereby the thicker, more consistent and denser coatings produced using the 10% TEOS are superior to those produced using the 2.5% TEOS.

The differences in the coating structure and performance between the 2.5% and 10% TEOS indicate that there is a limitation to the concentration of TEOS that can be used to produce homogeneous, dense coatings on PU substrates. Contact angle measurements, given in Figure 6, show that there is little difference in the contact angle of the 2.5% Si and 10% Si coatings at 13° and 9°, respectively, when applied to a PU substrate. This shows that the wetting of the PU surface is not an issue for either coating.

The chemical composition of the two TEOS concentrations when cured on a PU substrate was analysed using ATR-FTIR, which is shown in Figure 11. The signal response for the coating produced using the 2.5% TEOS is poor, and peaks are not observed compared to those of the coating produced using the 10% TEOS. This poor response is further evidence that the concentration of Si species provided by the 2.5% TEOS is not high enough to produce a homogeneous coating.

## 5. Conclusions

This paper investigated the feasibility of TEOS-based sol–gel coatings to be used as an overcoat to a polyurethane-coated steel surface and determined whether they offer corrosion protection. Sol–gel coatings, based on TEOS, were formulated to provide either a 2.5% Si or 10% Si oxide yield and applied via spray coating. Corrosion performance was determined via salt spray testing and the coatings were characterised to determine the effect of coating morphology and formation on the observed performance. The findings were as follows:

- It is possible to overcoat a PU-coated steel panel using a TEOS-based sol–gel. The optimisation of the oxide film application can facilitate the production of high-quality coatings even with a low concentration of TEOS and a short exposure time. Coatings produced using the 10% TEOS were dense, homogeneous and thicker than those produced using the 2.5% TEOS. Salt spray testing showed that these coatings provided substantial improvements in corrosion resistance with no scribe creep observed and a reduction of up to 21% edge creep compared to that of an uncoated PU substrate. This is suggested to be due to the sol–gel coating providing a barrier to oxygen and thereby reducing cathodic disbondment.
- Coatings produced using the 2.5% TEOS were inhomogeneous and had a rough morphology. The low concentration of Si species available to undergo hydrolysis and condensation at the PU surface limits the ability to form a consistent coating. Island-like formation, similar to that of the coffee-ring effect, was observed. The poor coating formation reduced the effectiveness of the coating’s corrosion resistance when subjected to salt spray testing.
- A novel closed-loop reactor was used to determine the effect of the sol–gel coating to limit the diffusion of O_2_, which reduces the rate at which CO_2_ evolves from the UV degradation of a PVB coating containing TiO_2_. This technique gave further evidence that the 2.5% TEOS afforded a poor barrier performance compared to that of the 10% TEOS, which can be attributed to inhomogeneous coating formation.
- ATR-FTIR analysis of the coatings showed that there was a significant reduction in Si–O–Si vibrations recorded for a coating produced using the 2.5% TEOS compared to those of the 10% TEOS, suggesting the reduced development of the sol–gel network.

This work provides evidence that an improvement in corrosion resistance can be achieved to organically coated steels using a TEOS-based sol–gel overcoat. The results show that there is a limit to the minimum concentration of TEOS that can be used to produce a dense, homogeneous coating that can act as a gas barrier for a PU substrate. The use of sol–gel coatings for coil-coating applications offers the ability to reduce material usage, CO_2_ footprint and volatile organic compounds [51]. These TEOS-based sol–gel coatings could find use as a durable, low-cost and environmentally friendly alternative to fossil fuel-based coating systems. Future work is required to determine the feasibility of the processing conditions required to produce the optimal coating and to determine if other sol–gel systems are more suitable for use as an overcoat to polymeric surfaces.

## Figures and Tables

**Table 1:** Sol–gel coating parameters.

Sample I.D.	SiO_2_ Yield (wt.%)	Residence Time (s)	Dosage (µL/µm^2^)
A	2.5	0.72	1.11
B	2.5	1.44	2.22
C	2.5	2.88	4.44
X	10	0.72	1.11
Y	10	1.44	2.22
Z	10	2.88	4.44

**Table 2:** Coating thickness and roughness data for sol–gel-coated PU samples provided by SEM and WLI data.

Sample I.D.	Average Coating Thickness (µm)	Surface Roughness (Ra) (µm)
Uncoated PU	-	0.75 ± 0.12
A	4.6 ± 0.3	0.7 ± 0.05
B	4.3 ± 0.6	3.69 ± 0.32
C	5.1 ± 0.7	3.1 ± 0.98
X	4.7 ± 0.3	0.72 ± 0.04
Y	5.8 ± 0.2	0.59 ± 0.04
Z	5.7 ± 0.2	0.72 ± 0.1

## Data Availability

The data used to support the findings of this study are included within the article and are available from the corresponding author upon request.
